# A List of the Diseases of the Patients Admitted at the Public Dispensary, in Lancaster, in the Year 1805

**Published:** 1806-08

**Authors:** 


*A List of the Diseases of the Patients admitted at the Public Dispensary, in Lancaster, in the Year* 1805.

| | | | |
|---|---|---|---|
| Ague . . . . . . . . | 13 | Female Complaints . | 43 |
| Fevers . . . . . . . | 101 | Epilepsy . . . . . . . | 5 |
| Measles . . . . . | 100 | Nervous Complaints . . | 26 |
| Small-pox . . . . . | 67 | Mental Derangement . | 1 |
| Cow-pox by Inoculation | 35 | Stomach Complaints . | 93 |
| Erysipelas . . . . . . . | 5 | Worms . . . . . . | 55 |
| Diseases of the Eyes . | 54 | Bowel Complaints . . | 61 |
| Diseases of the Mouth | 28 | Hæmorrhoids . . . . | 12 |
| Quinsy . . . . . . . . | 2 | Jaundice . . . . . . . | 6 |
| Croup . . . . . . . . | 4 | Dropsy . . . . . . . | 17 |
| Catarrhal Affections . | 133 | Rickets . . . . . . . | 8 |
| Hooping Cough . . . | 27 | Scrofulous Affections . | 31 |
| Asthma . . . . . . . | 41 | Scald Head . . . . . | 7 |
| Consumption . . . . . | 19 | Ulcers, &c. . . . . . | 39 |
| Pleurisy . . . . . . . | 14 | Cutaneous Eruptions . | 91 |
| Acute Rheumatism . . | 10 | Sprains and Bruises . | 56 |
| Chronic Rheumatism . | 50 | | |
| Calculous Complaints . | 24 | Total | 1278 |

| | |
|---|---|
| Cured . . . . . . . . . . . . . | 1162 |
| Relieved . . . . . . . . . . . | 50 |
| Died . . . . . . . . . . . . . | 37 |
| Remaining . . . . . . . . . . | 29 |
| Total | 1278 |

